# Algorithm-Based Risk Identification in Patients with Breast Cancer-Related Lymphedema: A Cross-Sectional Study

**DOI:** 10.3390/cancers15020336

**Published:** 2023-01-04

**Authors:** Mauro Nascimben, Lorenzo Lippi, Alessandro de Sire, Marco Invernizzi, Lia Rimondini

**Affiliations:** 1 Center for Translational Research on Autoimmune and Allergic Diseases-CAAD, Department of Health Sciences, Università del Piemonte Orientale “A. Avogadro”, 28100 Novara, Italy; 2 Enginsoft SpA, 35129 Padua, Italy; 3 Physical and Rehabilitative Medicine, Department of Health Sciences, Università del Piemonte Orientale “A. Avogadro”, 28100 Novara, Italy; 4 Infrastruttura Ricerca Formazione Innovazione (IRFI), Azienda Ospedaliera SS. Antonio e Biagio e Cesare Arrigo, 15121 Alessandria, Italy; 5 Physical and Rehabilitative Medicine Unit, Department of Medical and Surgical Sciences, University of Catanzaro “Magna Græcia”, 88100 Catanzaro, Italy

**Keywords:** breast cancer, lymphedema, medical algorithm, machine learning, dimensionality reduction, precision medicine, decision support system, prognostic map

## Abstract

**Simple Summary:**

The current study employed a cohort of 294 patients from two hospitals in northern Italy initially assembled to highlight factors leading to one consequence of breast cancer (BC): upper limb unilateral lymphedema (BCRL). BCRL occurrence is a multi-factorial pathological condition that is not widespread, with a medium-long-term onset affecting not only physical function but also the quality of life of BC survivors. In the current study, we employed the data to stratify the risk of BCRL using unsupervised low-dimensional data embeddings and clustering. In the proposed approach, the ordinal and the binary patients’ clinical variables were modeled separately in two distinct embeddings. Afterward, they were merged; thus, the final representation was a single prognostic map displaying three clusters of patients with peculiar features. The characteristics of each group were extracted and evaluated, identifying the factors associated with the high-risk cluster. Our findings might provide future insight into a precise risk stratification to target high-risk patients with tailored therapeutic intervention and focus resources on patients who deserve more attention.

**Abstract:**

**Background:** Breast cancer-related lymphedema (BCRL) could be one consequence of breast cancer (BC). Although several risk factors have been identified, a predictive algorithm still needs to be made available to determine the patient’s risk from an ensemble of clinical variables. Therefore, this study aimed to characterize the risk of BCRL by investigating the characteristics of autogenerated clusters of patients. **Methods:** The dataset under analysis was a multi-centric data collection of twenty-three clinical features from patients undergoing axillary dissection for BC and presenting BCRL or not. The patients’ variables were initially analyzed separately in two low-dimensional embeddings. Afterward, the two models were merged in a bi-dimensional prognostic map, with patients categorized into three clusters using a Gaussian mixture model. **Results:** The prognostic map represented the medical records of 294 women (mean age: 59.823±12.879 years) grouped into three clusters with a different proportion of subjects affected by BCRL (probability that a patient with BCRL belonged to Cluster A: 5.71%; Cluster B: 71.42%; Cluster C: 22.86%). The investigation evaluated intra- and inter-cluster factors and identified a subset of clinical variables meaningful in determining cluster membership and significantly associated with BCRL biological hazard. **Conclusions:** The results of this study provide potential insight for precise risk assessment of patients affected by BCRL, with implications in prevention strategies, for instance, focusing the resources on identifying patients at higher risk.

## 1. Introduction

Due to the increasing overall survival of breast cancer patients and the consequent increase in BC survivors, a growing interest has been raised in disabling the consequences of cancer and its treatment [1,2,3,4,5,6,7,8,9,10]. Breast cancer-related lymphoedema (BCRL) is one of the most common chronic disabling disorders that might affect over 50% of BC survivors [11,12,13]. It is characterized by localized tissue swelling associated with fluid retention related to surgical procedures and/or radiotherapy in breast cancer (BC) patients [14]. BCRL might often lead to psychophysical frailty with detrimental consequences on work, career, and Health-Related Quality of Life (HR-QoL) [15,16,17,18]. Despite the disabling consequences of BCRL being widely documented, few guidelines are currently available, and the optimal management of BCRL is still challenging [19,20,21,22]. Moreover, recent reports confirmed that BCRL is still regrettably underdiagnosed and undermanaged with heterogeneous therapeutic approaches in prevention and treatment plans, which severely vary between different institutions and countries [23,24]. On the other hand, the increasing number of long-term BC survivors emphasized the need for effective preventive strategies to address the survivorship issues better [25]. In more detail, Lin et al.’s recent meta-analysis of randomized controlled trials [26] underlined that patients treated with manual lymphatic drainage (MLD) have a lower incidence of lymphedema (RR = 0.58, 95% CI [0.37, 0.93], *p* = 0.02). Similarly, the systematic review by Hayes et al. [27] highlighted the significant effects of exercise therapy in preventing BCRL (RR = 0.49, 95% CI [0.28, 0.85]). However, there is still a lack of consensus about the precise identification of patients at higher risk, and there are no effective predictive tools to focus resources on rehabilitation plans to prevent BCRL, reducing its disabling consequences in BC survivors [26]. In this scenario, growing attention has been recently raised to machine learning solutions in BC management, with promising implications in developing self-improving technological models to guide clinicians in a precision medicine approach [28]. Interestingly, in 2018, Fu et al. [29] realized and validated a real-time diagnostic tool for BCRL, assessing the most common symptoms mentioned by BCRL patients. In more detail, the authors assessed a 26-item tool assessing self-reported symptoms, integrating a novel machine learning algorithm in the diagnostic process of BCRL to promote an early and time-efficient detection of lymphedema status. Recently, Wei et al. [30] developed a machine learning algorithm based on 24 items and included lymphedema symptoms assessment to diagnose lymphedema. Despite the positive results of these studies, self-reported symptoms for patients with BCRL might be affected by the intrinsic limitation of individual subjectivity that might crucially affect machine learning algorithms [31,32]. Moreover, to the best of our knowledge, the currently available literature on machine learning mainly concentrate on diagnostic tools without focusing on the prevention of BCRL based on the intrinsic characteristics of BC patients, including both cancer characteristics and cancer treatments. These findings underlined the need for practical preventive tools to close the gap between BC survivors and preventive, therapeutic programs, integrating technological advances and machine learning algorithms in the comprehensive management of BC survivors with a high risk of BCRL. Although several risk factors have been identified in BCRL onset [10,15,33], their synergisms in BCRL development have not been deeply studied yet. In order to better assess the multilevel interactions between different variables, machine learning might formulate complex models integrating artificial intelligence to characterize the latent structures between the input variables [34,35]. In this scenario, Uniform Manifold Approximation and Projection (UMAP [36]) is a dimensionality reduction algorithm recently applied in medicine and genomics [37,38,39], able to preserve the structure of relations in the data. UMAP assumes the input data are uniformly distributed on a Riemannian manifold [40], a topological space capitalizing on the local linearity found in manifolds retaining local neighborhoods. Fundamentals features of Riemannian manifolds are the ability to define angles and lengths over curves of the manifold. To assess this task, a metric that is constant over the manifold to preserve its structure should be chosen. This step also influences the construction of the simplicial complexes grouping a certain number of neighbor points. These sets of simplicities capture the initial underlying topology of the dataset as a weighted graph. UMAP solves a minimization problem, usually employing cross-entropy and stochastic gradient descent to lower the dataset’s dimensionality so that the high-dimensional dataset and the low-dimensional projection are analog in terms of probabilistic similarity. The UMAP bi-dimensional representations of multi-dimensional datasets are dense point clouds that are easy to visualize, cluster, and interpret. In light of these considerations, UMAP might have a role in developing predictive tools that might guide clinicians in the tailored prescription of preventive rehabilitation plans. Therefore, this cross-sectional study aimed to characterize the risk of BCRL by autogenerated clusters of BC patients, extracting relevant patterns or factors from the unsupervised dimensionality reduction achieved with the UMAP technique.

## 2. Materials and Methods

The present study analyzed a multi-centric dataset from two northern Italy hospitals containing clinical information about BC female patients. The set of data comprised clinical factors from women who had undergone axillary dissection for BC, gathering twenty-three clinical variables and metadata from anonymized subjects. Patients’ clinical status was assembled over a period ranging from January 1998 to September 2018. The list of variables representing the clinical status of each patient included in the study was reported in Table 1 and detailed in Table A1. The clinical variables have been investigated through UMAP employing a novel approach fusing heterogeneous attributes. This methodology produced a non-linear dimensionality reduction of the initial database aiming to define hidden relations in medical files not directly observable in the original records and influencing BCRL incidence.

The variables considered included patients characteristics (AGE, BMI, NCD, and HR DRUG), macroscopical cancer features (SIDE, G, T, N, and NR METASTATIC LN), anatomopathological cancer attributes (HISTOTYPE, MOLECULAR SUBTYPE, ER, PR, HER2, Ki67, LVI, and ECE), surgical therapies (BREAST SURGERY and TOTAL NR DISSECTED LN), and medical therapies (RT TYPE, TAXANE BASED CT, HT, and TTZ). Lymphedema-related measurements, for example, limb volume changes, were not considered.

Among them, 9 were ordinal or categorical variables (NR METASTATIC LN, TOTAL NR DISSECTED LN, RT TYPE, HR DRUG, HISTOTYPE, G, T, N, and MOLECULAR SUBTYPE). At the same time, the remaining 12 were binary values (BREAST SURGERY, SIDE, Ki67, TAXANE BASED CT, HT, TTZ, LVI, ECE, ER, HER2, NCD, and PR). AGE and BMI were continuous data transformed into ordinal variables by binning the values into ten ordinal levels and renamed AGE GROUP and BMI GROUP. Categorical variables were converted into numerical values. Later on in the text, non-ordinal categorical, ordinal, and continuous converted to ordinal clinical variables will be called ordinal variables except where otherwise indicated. Table A1 of Appendix A details information about each variable included in the dataset. Binary variables have two levels and could mean presence or absence in the case of 0 or 1, or two types (i.e., for SIDE, 1 means left and 2 means right). The column “Levels” in Table A1 could be intended as the number of unique values.

**Figure 1:**
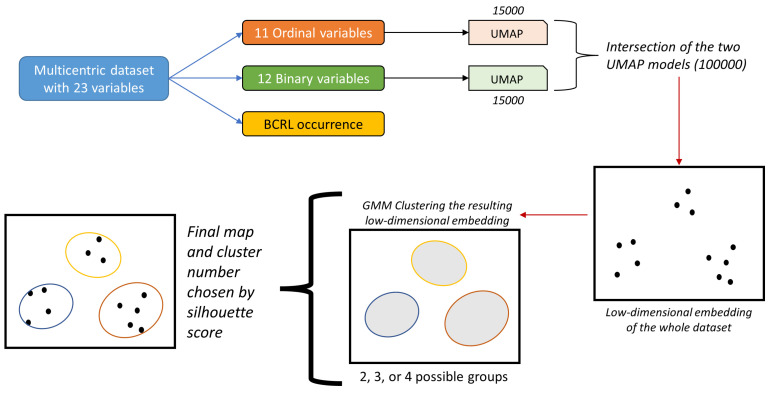
Overview of the procedure leading to patient grouping.

UMAP tries to preserve the local and global information contained in the input variables by capturing the latent structures of the initial high-dimensional dataset and representing them as a visualizable graph. This feature is one fundamental difference between UMAP and another well-known dimensionality reduction technique, Barnes-Hut-SNE, that preserves only the local data structure, as previously investigated by the same authors [41]. Preserving the entire initial data structure allows for adding new data to a learned representation. Moreover, UMAP supports merging distinct models by intersection, union, or subtraction. In the current investigation, we leveraged both properties of UMAP. In the proposed approach, the ordinal and the binary variables were modeled separately in two distinct UMAP models. After obtaining the two UMAP models, the final representation was a single low-dimensional embedding that merged the two UMAP graphs by intersection. The current study coded UMAP to produce bi-dimensional charts with axes representing two UMAP projections that summarize the whole dataset over a two-dimensional plane. This analysis exploited several advantages of the UMAP technique: one is to overcome euclidean distance limitations in high dimensions and use other metrics between nearest neighbor points. In this way, the manifold’s local connectivity is guaranteed. The number of nearest neighbors to build the graph is not determined automatically by the algorithm but by users, together with the “minimal distance” parameter that, acting on the curves defining the distance probability between points, induced low-dimensional dense clouds of values. Parameter space investigation was performed by random search over a grid of 1,422,960 possible parameters for the ordinal and 1,164,240 for the binary variables. Initially, 15,000 UMAP models were prepared for the binary and ordinal sets, whereas 100,000 random combinations derived from these models were evaluated as the final map. The final map aimed to produce a low-dimensional embedding of the patients that facilitated their categorization (or labeling) in groups associated with different BCRL risk profiles. The whole experimental procedure is exemplified in Figure 1.

The final bi-dimensional graph represented a low-dimensionality embedding of the original dataset over a unitless Cartesian plane. A Gaussian mixture model (GMM, [42]) determined the number of clusters: the initial GMM parameters were identified by the k-means algorithm and later tuned with a Gaussian probability distribution. For each bi-dimensional map, three configurations were built with two, three, or four GMM clusters and evaluated by silhouette score. Only clusterings with a silhouette score of at least 0.6 were retained, and upon visual inspection, the model with the highest silhouette score was selected as the final low-dimensional embedding. This final UMAP map joined the two separate UMAP models obtained from the ordinal or binary variables, whose parameter sets were included in Table 2. The silhouette score of the current final map was 0.805.

**Figure 2:**
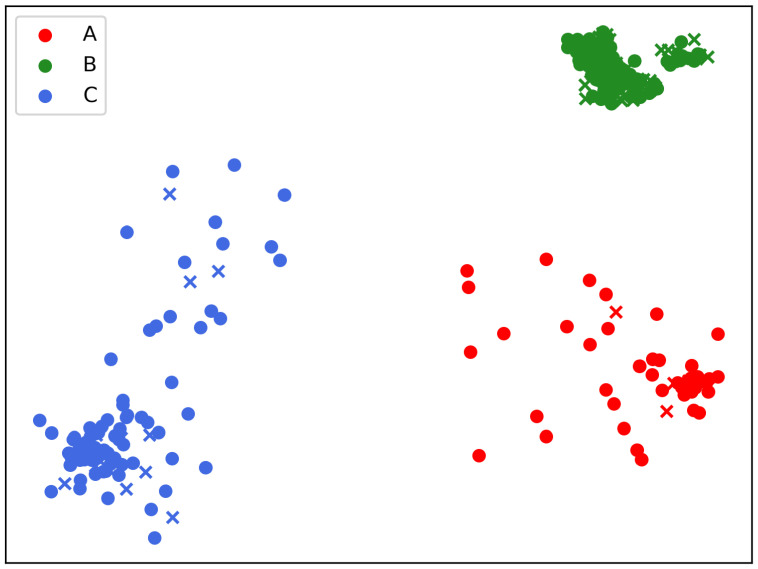
Low-dimensional embedding of the patients into a bi-dimensional map: each point is a patient colored according to clustering into the three groups A, B, and C. In the above figure, *dots* depict patients without BCRL, while *crosses* represent patients with the disease.

The next section of the study investigated the characteristics of the final map after clustering the patients to extract relevant patterns or factors present in the groups created by the unsupervised visualization. The UMAP bi-dimensional points representing patient data were labeled as belonging to A, B, or C clusters (Figure 2). Indeed, the number of clusters was in accordance with the presence of three dense and well-separated groups on the final map. Given that each image point depicted a patient, applying the proposed procedure could be relevant in highlighting patterns or finding hidden relations among initial variables. As a side note, the presence of well-defined and dense data groups on the final graph ensured that BCRL patients and cluster membership were mutually exclusive.

The clustering procedure found that each label (A, B, and C) is associated with a different risk profile of BCRL occurrence. In particular, clusters A and C have lower percentages of BCRL patients than cluster B (Table 3). Table 3 reports the absolute number of patients in each cluster and their percentages.

The value counts in Table 3 suggested the presence of “order” among clusters, which could be reorganized as in the following Table 4, grading the probability of BCRL occurrence. In this way, the clusters could be interpreted as explanatory ordinal variables with three categories associated with the percentages of patients suffering from BCRL.

### 2.1. Alternative Clustering into Two BCRL Risk Groups

**Figure 3:**
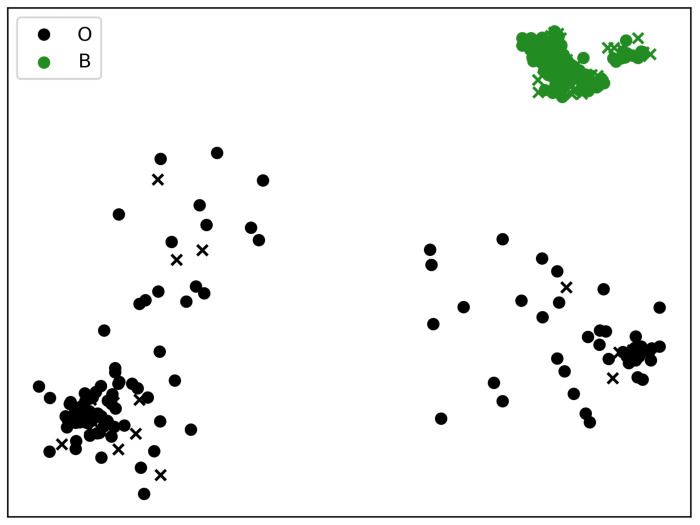
The patients in clusters A and C were merged into a new cluster named O. In the above image, *crosses* are BCRL patients, while *dots* are subjects without the disease.

Table 4 reports the absolute number of patients in each cluster: considering the number of patients without BCRL in A and C summed to 118, this was closer to the number of patients without the disease as found in cluster B. Under this perspective, it could be advisable to have groups of patients with a nearly balanced number of negative cases to assess BCRL risk. In this way, the comparisons might be meaningful because equalizing the number of negative subjects could highlight different drivers or hidden factors in BCRL prognosis. It also facilitates the application of machine learning techniques as supplementary methods for data investigation. Cluster B collects 53% of the patients in the dataset; thus, clusters A and C were joined in a unique group, gathering nearly 47% of the remaining subjects. This operation led to the creation of a new cluster O (aka “Others”), gathering all subjects from A and C, as shown in Figure 3 and Table 5.

The new distribution of patients among clusters B and O are shown in Table 5

In Table 5, clusters O and B contain nearly the same negative cases but a different proportion of BCRL positives. The values of the variables determining cluster B membership might be interpreted as “high risk” in exhibiting the presence of the disease. In contrast, biomarkers leading to the classification of a patient into cluster O might be associated with a lower probability of BCRL positivity. In the original data, the BCRL point prevalence was ($\frac{70}{294}$) 23.81%, slightly above the population frequency estimated from the literature ranging from 16.6% [43] to 20% [15]. Cluster O has a point prevalence of ($\frac{20}{138}$) 14.5%, while cluster B of ($\frac{50}{156}$) 32.05%. The prevalence ratio between clusters is 2.2, revealing that, in cluster B, the prevalence is more than double.

## 3. Results

The dataset comprised clinical information on 294 women, 70 affected by BCRL and 224 without BCRL (mean age: 59.823±12.879 years, from Table A1). The average BCRL occurrence was 854.85 days (equivalent to 2 years and four months). The presentation of the analysis results was divided into three sections: statistics and risk profiles obtained from the three clusters characterization of the dataset (A, B, C) in Section 3.1, statistics and risk profiles obtained from the two clusters labeling of patients (clusters B and O) in Section 3.2, and machine learning evaluation of a possible scheme to automatically label patients into three or two risk categories in Section 3.3.

### 3.1. Statistics on Three Clusters (A, B, C)

The Chi-Square test on the contingency table (Table 4) as a measure of association rejects the null hypothesis of no association (or independence) between the variables ($\chi^{2}=13.601,p=0.001113$). Consequently, the proportion of BCRL in patients is dependent on the categorization into A, B, and C labels; in other words, BCRL occurrence is not equally distributed across clusters, and each cluster might be associated with a different risk of BCRL development. However, the association is not strong, probably due to imbalanced data because patients without BCRL are more than three times those with BCRL; indeed, the Cramer’s V coefficient is 0.2151. Assuming one degree of freedom, a Cramer V of 0.3 could be interpreted as a medium association: the obtained value of 0.21 could be evaluated as a mild association between cluster assignment and BCRL presence. At the two-sided Fisher’s Exact test using the Freeman–Halton extension, the hypergeometric probability that clusters are equally likely to gather BCRL patients is 0.09473%, thus below the significance level of 5%. This additional statistical proof sustains the possibility of dependence between BCRL counts and cluster membership. The re-organization of Table 2 into Table 4 promoted the conduction of the Cochran–Armitage trend test (Z=3.643,p=3× 10${}^{-4}$) and confirmed the presence of a linear trend in the contingency table.

From Table 4, it could be possible to calculate the probabilities that a patient chosen from the sample is affected by BCRL and belongs to clusters A, B, or C (Table 6), keeping in mind that the overall marginal probability of BCRL is 23.8%.

Cluster B gathers the highest percentage of BCRL patients, with a conditional probability above the total marginal likelihood of having developed BCRL. Future patients inserted in this cluster might have a higher chance of having BCRL based on the patient’s variables. Note that the “Conditional Probability” is equivalent to the “Point Prevalence”, whereas the “Joint Probability” can be interpreted as the “Incidence Rate” over the observed period needed to collect the dataset. The second row of Table 6 could be interpreted as the risk of BCRL occurrence inside each cluster. In contrast, the third row of Table 6 shows the probability that one patient suffering from BCRL will be categorized in each group by the algorithm proposed in the current investigation. These observations substantiated the hypothesis of considering cluster B as the “high risk” group for BCRL occurrence and the variables leading to categorizing a patient in this cluster as those most influential in BCRL determination.

Additionally, proportions of patients affected by BCRL and free from the disease were also reported in Table A14 of Appendix C. The rate ratio of BCRL inside groups is two times as high in B than in C and nearly five times in B compared to A.

### 3.2. Statistics on Two Clusters (B vs. “Others”)

In Section 2.1, clusters A and C were merged into a single cluster called “Others” and abbreviated as O. This operation produced two clusters (B and O) with a more balanced number of patients that are not exposed to the disease and total value counts were more balanced.

Statistics on the patient counts of the two-by-two Table 5 confirmed a significant association between clusters’ membership and BCRL outcome (association between rows and columns) established by Chi-square with Yates correction ($\chi^{2}=11.496,p=0.0007$). Cramer ϕ as a measure of effect size was 0.21. The proportion of patients belonging to O and having BCRL is 0.17, whereas the incidence proportion of patients in B suffering from BCRL is 0.47. Subjects included in cluster B had a 30% excess probability of suffering from BCRL compared to patients classified in cluster O.

#### 3.2.1. Ordinal and Categorical Variables Analysis

Statistical analysis was addressed by the Mann–Whitney U test between and within comparisons. Significant differences between cluster B (higher risk) and cluster O (lower risk) were found in the variables reported in Table 7. The table displayed significance if the tests were below p≤0.05. No significant differences were calculated during the within-cluster analysis.

The categorical (non-ordinal) variables were compared in terms of modal value; it is the most common value encountered in the distributions and described in Table 8.

The clinical factors prominent in categorizing a patient in cluster B or O and identifying BCRL occurrence are NR METASTATIC LN, HR DRUG, and AGE GROUP. These three components show the highest *p*-values at the statistical test or the most considerable difference between modes. Other relevant elements were G, N, and BMI GROUP.

#### 3.2.2. Binary Variables Analysis

Binary variables are those that assume precisely two values. During this analysis, two 2×2 frequency tables, one for cluster B and one for cluster O, were created for each binary variable. The goal was to compare the cumulative incidence of exposed groups in both clusters. The term “risk” used in the first two columns of Table 9 refers to the cumulative incidence of the patients with BCRL divided by the sum of patients exposed to the variable of interest. This formulation addressed the event rate of BCRL as an absolute risk difference (last column of Table 9). The risk difference could also be employed in frequency tables with zero entries and selected for this reason. In addition, it is a measure straightforward to interpret, showing the difference in risk between clusters.

**Figure 4:**
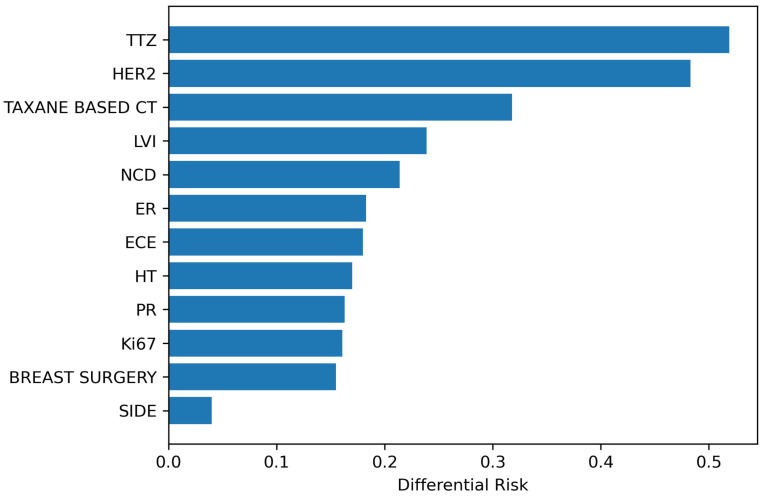
Binary variables’ absolute differential risk ordered by magnitude.

Binary variable values could be interpreted as whether a patient is exposed to a treatment or not; this holds except for SIDE, which means the body part affected by breast cancer. For the SIDE variable, the absolute risk difference calculation was less meaningful and resulted in balanced “risks” between clusters. Concerning the other variables, the top three differences between the risks associated with clusters were in TTZ, HER2, and TAXANE BASED CT variables (Figure 4). Further, two variables related to a moderate risk difference between clusters were LVI and NCD.

The association between BCRL occurrence and the outcomes of the binary variables was tested statistically for each cluster: Table A15 of Appendix D shows the variables where the statistical significance was below the threshold of p≤0.05 for the Fisher exact test (in the presence of small values in the frequency tables) or the $\chi^{2}$ test of independence. These tests found a relevant association between BCRL distribution and SIDE or TTZ (her2+) in cluster B and nearly significant in LVI (p=0.0628) and HER2 (p=0.0636).

### 3.3. Demonstration of Automatic Patient Categorization

Five machine learning models have been trained to demonstrate the possibility of employing cluster labels to categorize patients. This numerical experiment tested whether models can accurately label patients or not using the whole set of ordinal and binary variables. In previous sections, it has been shown how each label represented a different risk profile of being affected by BCRL. Suppose the 23 variables can be automatically related to the risk profiles generated by the procedure employing UMAP. In that case, in the future, it could be possible to categorize new patients automatically through a trained machine-learning model. The classifiers evaluated during this numerical experiment were Logistic Regression (LR), Random Forest (RF), Linear Discriminant Analysis (LDA), Naive Bayes classifier, Adaptive Boosting Classifier (ABC), and Randomized decision trees (ET). The six classifiers were selected due to their different ability to handle heterogeneous input variables. LR is a statistical machine learning classifier that handles natively binary and categorical qualitative data, performing well on binary outcomes (e.g., when two clusters were the output). LDA finds a linear combination in the input features through a discriminant function: it is more suited than LR to classify multiclass outputs (e.g., when three clusters were the variable to be predicted [44]). RF, ET, and ABC are ensembles of decision trees, a classic data mining algorithm [45]. Ensemble learners generally have a superior capability of modeling complex input data compared to single decision trees because aggregated classifiers perform better than single ones [46]. RF performs bagging without assumptions regarding input data distribution (which is why support vector machines were not included), and using random feature splits can afford highly dimensional datasets. ET is similar to RF but does not achieve bootstrapping and might be computationally faster, offering a term of comparison to RF in the case of noisy features. ABC works on boosting rather than bagging and adaptively weights hard-to-classify samples integrating a different voting mechanism when selecting the outcome class [47]. The Naive Bayes Classifier reduces input features (categorical or numerical using thresholds) to binary decisions and might have a good performance on a dataset with mixed variable types [48].

The best model has been selected by repeated stratified cross-validation (5-folds CV with ten repetitions) and used to evaluate the classifiers’ performance on the current dataset. It should be underlined that classifiers were not optimized and were left with their default hyperparameters as implemented in [49]. Skipping the hyperparameter optimization phase was decided to judge the ability of the classifiers in their basic form and without introducing bias for one or another; consequently, CV outcomes could be evaluated to assess estimator performance only. Additionally, classes were weighted to account for eventual unbalances in the number of instances.

**Figure 5:**
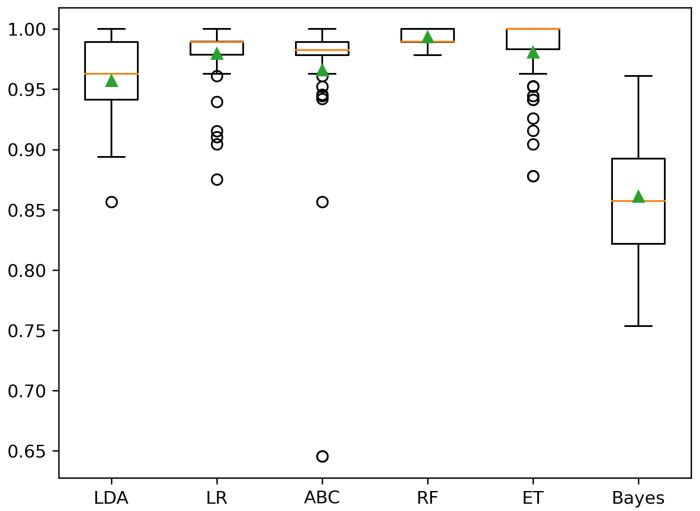
Balanced accuracy at CV using three labels (A, B, C).

**Figure 6:**
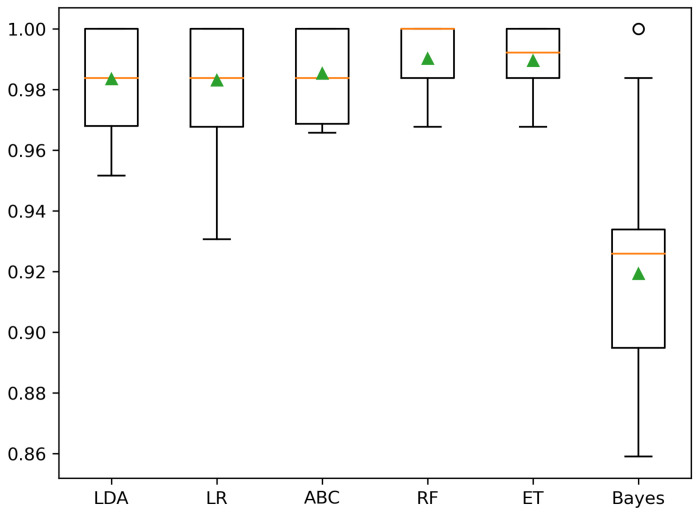
Balanced accuracy at CV using two labels (B, O).

The results of this section were quite promising in establishing a model able to connect the 23 patient variables with the labels obtained by clustering the UMAP-derived low-dimensional map. With three labels (A, B, and C), the peak performance is achieved by an RF classifier (balanced accuracy 99.4±0.7%), whereas using two labels (B and O) to categorize the patients, the best classifier is shared by ET (balanced accuracy 99.0±1.2%) and RF (balanced accuracy 99.0±1.2%). In both situations, other classifiers reached comparable accuracies, as shown in the boxplots in Figure 5 and Figure 6. Table 10 details each classifier’s balanced accuracies at CV, including a Dummy classifier employed to show the chance level.

## 4. Discussion

In the past few years, increasing interest has been rising in machine learning technology, addressing the challenge of guiding clinicians to precisely prescribing optimal treatments. In patients with BCRL, rehabilitation might improve the long-term management of their condition [26,27]; however, there is still a large gap in the knowledge about preventive rehabilitation strategies in patients with higher risk, and no guidelines characterize patients requiring this treatment. Moreover, effective BCRL predictive tools are lacking, and sustainable strategies focusing resources on patients at higher risk of BCRL are still challenging.

In the current manuscript, a novel procedure has been tested, employing a set of mixed variables (continuous, ordinal, categorical, and binary) to classify patients retrospectively. Two UMAP models were merged together, and this approach is uncommon in the previous literature, where UMAP was employed to model the data directly [50]. The clustering results identified three groups of patients, with a different number of BCRL patients occurring inside each group. Upon investigating two clusters to balance negative cases (Section 3.2), the factors leading to patient classification could be associated with a different probability of being affected by BCRL. Interestingly, our findings showed that the most influential variables associated with BCRL were NR METASTATIC LN, G, HR DRUG, AGE GROUP (ordinal set), TTZ, HER2, and TAXANE BASED CT (binary set). Other relevant clinical factors were N, BMI GROUP, LVI, and NCD. In cluster B, TTZ at the $\chi^{2}$ test (and partially LVI and HER2) had outcomes dependent on BCRL incidence (Appendix D, Table A15). These outcomes are consistent with what is emerging in the BCRL literature. In [51], the authors identified HER2 as a factor increasing BCRL risk, and TAXANE BASED CT being associated with TTZ treatment is another element crucial in BCRL occurrence. In another work [10], LVI was described as an indicator of BCRL. The number of lymph nodes with metastasis (NR METASTATIC LN) was already identified as a highly influential factor in BCRL onset [18,52], together with weight variations and obesity measured by BMI [53,54]. Evidence that secondary lymphedema is aggravated by hormone therapy was argued in [55]; indeed, during the present investigation, HR DRUG resulted in another distinguishing factor between patients of the two clusters. Low physical activity and younger AGE were related to functioning and HR-QoL [56]: some authors recognized an active role of AGE [57,58,59] as a BCRL risk factor; in contrast, others found less contribution of this variable to BCRL management [60]. Among BCRL predictors, breast tumor grade and lymphatic spread (G and N) were considered risk factors for BCRL in multiple sources from the medical literature [52,61,62]. In addition, the variables AGE, G, and N were also considered prognostic factors for survivorship [63]. Further, comorbidities (NCD), such as dyslipidemia [64], and diabetes [59], might exacerbate BCRL, especially in aged patients. To summarize, lymphedema post-breast cancer is a multi-factor disease with etiology not wholly understood; accordingly, the present analysis identified a subset of factors relevant to patient risk stratification based on cluster characteristics.

**Figure A1:**
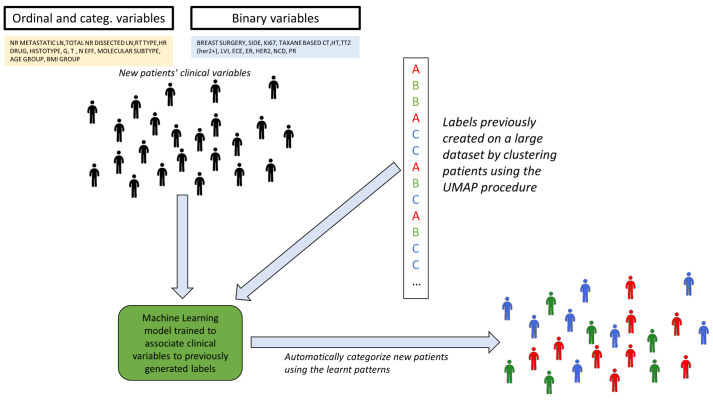
Possible usage of the proposed methodology in prospective studies.

The labels derived from clustering (A, B, and C or B and O) employing the novel methodology of merging two UMAP low-dimensional representations have been adopted to classify patients from the initial set of 23 clinical variables (Section 3.3). Five machine learning models were trained to categorize the patients by connecting the 23 variables to the cluster labels created by the UMAP methodology. Machine learning techniques showed high accuracy in determining patient labeling, opening the way for future investigations on employing the proposed procedure in precision medicine settings. The high performance obtained meant a machine learning algorithm could quickly establish a connection between clinical variables and the cluster labels and possibly apply those labels to new patients, as illustrated in Figure A1 of Appendix E.

To the best of our knowledge, this is the first study integrating a machine learning technology in precisely assessing BCRL patients. The results of the present study might guide clinicians in the tailored management of BC patients based on objective data; in particular, a precise clusterization might identify patients at high risk, guiding the prescription of preventive strategies to reduce lymphedema onset and optimizing resources [64,65]. On the other hand, in patients with medium risk, a closer follow-up might be proposed to optimize patient monitoring or early rehabilitation treatment [17,66]. However, it should be noted that this is not the first application of machine learning in the BCRL field. In particular, the previous study by Wei et al. [30] developed a web-based machine-learning algorithm to improve the real-time monitoring of symptoms mentioned by BC survivors. On the other hand, the logistic regression showed good sensitivity and specificity only in BCRL diagnosis, without focusing on risk assessment and patient risk stratification. Similarly, the study by Fu et al. [29] developed a logistic regression model algorithm for the early diagnosis of BCRL. However, the authors considered only subjective symptoms without focusing on patients’ or cancers’ intrinsic characteristics. Moreover, no predictive algorithm was developed in that study, and no machine learning technology assessed the multilevel interactions among different variables. Therefore, no previous study has assessed the BCRL onset in BC survivors applying UMAP technology, considering the patients and intrinsic cancer characteristics to improve early detection and identify a precise risk stratification in clusters.

Another advantage of the proposed procedure is the possibility of creating bi- dimensional maps showing patient positioning in the embedded space (Figure 2 and Figure 3). Within this visualization, the plane’s regions might be associated with BCRL risk, creating an easily interpretable graph. The image areas related to the clusters in Figure 2 and Figure 3 could be delimited by boundaries and help clinicians visually illustrate the machine learning outcomes using graph-based intelligible health care models. Indeed, one criticism connected with machine learning in medicine is the lack of appealing explanations of artificial intelligence models [67].

Interestingly, the biomarker distribution using the unsupervised learning approach and two cluster mapping presented significant intra-cluster differences. In more detail, patients without BCRL and patients with BCRL in the two clusters were characterized by significant differences in age, grading, tumor local extension, and molecular subtype. The results of this machine learning clustering align with previous studies, underlining that these factors significantly impact the risk of lymphedema development [10,33,68,69]. Thus, recent research emphasizes that precise identifications of individual risk factors should be integrated into routine clinical practice to optimize a patient-centered approach targeting BCRL prevention [18]. In contrast, significant differences in terms of BMI were identified only in the BCRL patients, suggesting that the machine learning model might consider the body composition in the patient’s clusterization less important. Further studies are needed to clarify the role of BMI in a comprehensive risk assessment of patients with BCRL. However, our data suggested that it should be considered an essential coadjuvant in the lymphedema development of BC patients, in line with the current literature [54,70]. On the other hand, it is surprising that significant differences between clusters regarding cancer treatments were reported only in patients without BCRL. It might partly be due to the widely recognized role of surgery and radiotherapy in lymphedema onset [70,71], which might affect the BCRL development and, consequently, patients clusterization, but require other potential interactions with different variables in patients with BCRL.

In conclusion, this multi-centric cross-sectional study developed a novel methodology integrating several variables for BC patients’ risk stratification, providing different clusters addressing the multilevel interaction of the most common risk factors for BCRL. However, it should be noted that this cross-sectional study is not free from limitations. First, all the patients considered underwent axillary dissection. Indeed, most available studies considered axillary dissection the most important risk factor for BCRL [33,72]. Lastly, the retrospective data might provide information only about the potential association between the different variables used for patients’ clusterization. Therefore, future prospective studies are necessary to better characterize the effects of integrating this novel machine-learning algorithm in clinical settings.

## 5. Conclusions

To date, effective predictive tools for BCRL are an urgent need in the current literature due to the growing prevalence of BC survivors. Our results provided evidence about a novel procedure addressing the multilevel interactions between 23 common risk factors involved in BCRL onset. The clusters developed by UMAP might guide clinicians in a precision medicine approach to tailor preventive strategies to individual risk. Future research might further improve our artificial intelligence model to better characterize the role of different variables in reducing BCRL onset and improving long-term management of BC survivors through clinical status prediction.

## Figures and Tables

**Table 1:** Variables included in the study.

Variable	Type	Description
NR METASTATIC LN	Ord.	Number of metastatic lymph nodes
TOTAL NR DISSECTED LN	Ord.	Number of dissected lymph nodes
RT TYPE	Cat.	Types of radiation therapy (breast, supraclavicular fossa, chest wall)
HR DRUG	Cat.	Type of estrogen therapy before breast cancer
HISTOTYPE	Cat.	Characterization of lymph node histology
G	Ord.	Breast cancer grading
T	Ord.	TNM staging system: size or direct extent of the primary tumor
N	Ord.	TNM staging system: degree of spread to regional lymph nodes
MOLECULAR SUBTYPE	Cat.	Luminal A, Luminal B, ERBB2/HER2-amplified or Triple-negative
AGE	Cont.	Age of the patient at diagnosis
BMI	Cont.	Body Index Mass
BREAST SURGERY	Bin.	Type of breast surgery (quadrantectomy, mastectomy)
SIDE	Bin.	Side of breast cancer
Ki67	Bin.	Ki67 expression (low <18% or high >18%)
TAXANE BASED CT	Bin.	Underwent the Taxane-based Chemotherapy
HT	Bin.	Hormone therapy
TTZ	Bin.	Trastuzumab therapy
LVI	Bin.	Presence of Lymphovascular invasion
ECE	Bin.	Presence of Extracapsular Extension
ER	Bin.	Estrogen receptors
HER2	Bin.	Human Epidermal Growth Factor Receptor 2
NCD	Bin.	Presence of comorbidities
PR	Bin.	Progesterone receptor

**Table 2:** Parameters selected for the initial UMAP models.

Variable	Num of Neighbors	Learning Rate	Minimal Distance	Spread	Metric
Ordinal	44	0.0005	0.2	1.5	Canberra
Binary	38	0.5	0.99	3	Correlation

**Table 3:** Number of patients in each cluster and their percentages.

	Patients				Percentages %			
	**A**	**B**	**C**	**Margin**	**A**	**B**	**C**	**Margin**
Absence of BCRL	41	106	77	224	13.94	36.05	26.19	76.19
Presence of BCRL	4	50	16	70	1.36	17.0	5.44	23.80
Margin Total	45	156	93	294	15.3	53.0	31.63	100

**Table 4:** Patient distribution among clusters (re-ordered columns of Table 3).

	Cluster A	Cluster C	Cluster B	Margin Total
Presence of BCRL	4	16	50	70
Absence of BCRL	41	77	106	224
Margin Total	45	93	156	294

**Table 5:** Grouping patients from A and C into the new cluster O.

	Patients			Percentages %		
	**O**	**B**	**Margin**	**O**	**B**	**Margin**
Absence of BCRL	118	106	224	40.13	36.05	76.19
Presence of BCRL	20	50	70	6.8	17.0	23.80
Margin Total	138	156	294	46.93	53.06	100

**Table 6:** Probabilities of being a patient with BCRL given the categorization into three clusters.

	Cluster A	Cluster C	Cluster B
Joint Probability of BCRL among all patients	1.36%	5.44%	17.0%
Conditional Probability that a patient has BCRL	8.88%	17.2%	32.05%
given the patient belongs to cluster A, B, or C			
Conditional Probability that a patient suffering	5.71%	22.86%	71.42%
BCRL belongs to cluster A, B, or C			

**Table 7:** Significant differences in the Mann–Whitney U test B versus O between BCRL and patients without BCRL.

Variable	Clusters B vs. O	Clusters B vs. O
	Patients without BCRL	Patients with BCRL
NR METASTATIC LN	***	***
TOTAL NR DISSECTED LN	-	-
G	***	**
T	*	-
N	***	*
AGE GROUP	***	***
BMI GROUP	-	*

Legend: *p* ≤ 0.05: “*”, *p* ≤ 0.01: “**”, *p* ≤ 0.001: “***”, *p* > 0.05: “-”.

**Table 8:** Modal value of the categorical variables.

	Presence of BCRL		Absence of BCRL	
**Variable**	**Cluster O**	**Cluster B**	**Cluster O**	**Cluster B**
RT TYPE	1	1	1	1
HR DRUG	6	0	6	0
HISTOTYPE	1	1	1	1
MOLECULAR SUBTYPE	1	1	1	1

**Table 9:** Absolute risk difference between clusters.

Variable	Cluster B Risk	Cluster O Risk	Absolute Risk Diff.
BREAST SURGERY	0.257	0.102	0.155
SIDE	0.192	0.152	0.040
Ki67	0.337	0.176	0.161
TAXANE BASED CT	0.318	0	0.318
HT	0.316	0.146	0.17
TTZ	0.519	0	0.519
LVI	0.406	0.167	0.239
ECE	0.303	0.123	0.18
ER	0.328	0.145	0.183
HER2	0.483	0	0.483
NCD	0.354	0.14	0.214
PR	0.301	0.138	0.163

**Table 10:** Balanced accuracy of the tested classifiers at CV.

	Two Clusters (B, O)		Three Clusters (A, B, C)	
**Classifier**	**Mean BA (%)**	**SD BA (%)**	**Mean BA (%)**	**SD BA (%)**
Dummy	51.3	±5.3	32.5	±6
LDA	98.4	±1.5	95.7	±3.3
LR	98.3	±1.7	98.0	±2.6
ABC	98.5	±1.3	96.6	±6.9
RF	99.0	±1.2	99.4	±0.7
ET	99.0	±1.2	98.1	±3.2
Bayes	91.9	±3.3	86.1	±5.2

## Data Availability

Data are available upon reasonable request coming from verifiable email addresses. Code is available at https://github.com/m89p067/BCRL_unsup_clust, accessed 22 November 2022.

## Abbreviations

The following abbreviations are used in this manuscript:

ABC	Adaptive Gradient Classifier
BA	Balanced Accuracy
BC	Breast Cancer
BCRL	Breast cancer-related lymphedema
CV	Cross-Validation
ET	Randomized decision trees
GMM	Gaussian mixture model
HR-QoL	Health-Related Quality of Life
LDA	Linear Discriminant Analysis
LR	Logistic Regression
MLD	Manual lymphatic drainage
RF	Random Forest
RR	Relative Risk
SD	Standard Deviation
UMAP	Uniform Manifold Approximation and Projection

## Appendix A. Characteristics of the Clinical Variables

**Table A1:** Descriptive information of the variables included in the study.

Variable	Mean	Median	SD	SE	Levels	Range
NR METASTATIC LN	5.2959	2.0	7.2157	0.4208	33	[0, 37]
TOTAL NR DISSECTED LN	24.9626	24.0	8.6692	0.5056	42	[2, 58]
RT TYPE	1.1565	1.0	0.9997	0.0583	4	[0, 3]
HR DRUG	4.0442	5.0	2.1197	0.1236	6	[0, 6]
HISTOTYPE	1.4082	1.0	1.2652	0.0738	8	[1, 9]
G	2.3673	2.0	0.591	0.0345	3	[1, 3]
T	1.6803	1.0	0.8703	0.0508	4	[1, 4]
N	1.6054	1.0	0.8015	0.0467	3	[1, 3]
MOLECULAR SUBTYPE	1.8844	1.0	1.2143	0.0708	5	[1, 5]
AGE ^1^	59.823	61.0	12.879	0.7511	56	[26, 88]
BMI	26.926	26.03	5.8085	0.3388	264	[14.4, 57.2]
BREAST SURGERY	1.4048	1.0	0.4917	0.0287	2	[0, 1]
SIDE	1.4728	1.0	0.5001	0.0292	2	[1, 2]
Ki67	1.4184	1.0	0.4941	0.0288	2	[1, 2]
TAXANE BASED CT	0.5136	1.0	0.5007	0.0292	2	[0, 1]
HT	0.8537	1.0	0.354	0.0206	2	[0, 1]
TTZ	0.0918	0.0	0.2893	0.0169	2	[0, 1]
LVI	0.3571	0.0	0.48	0.028	2	[0, 1]
ECE	0.619	1.0	0.4864	0.0284	2	[0, 1]
ER	0.8741	1.0	0.3322	0.0194	2	[0, 1]
HER2	0.1088	0.0	0.312	0.0182	2	[0, 1]
NCD	0.6565	1.0	0.4757	0.0277	2	[0, 1]
PR	0.7925	1.0	0.4062	0.0237	2	[0, 1]

Legend: SD Standard Deviation, SE Standard Error. ^1^ Age at diagnosis.

## Appendix B. Binary Variables Cross-Tables

**Table A2:** BREAST SURGERY vs. BCRL contingency table.

BREAST SURGERY	BCRL (Unaffected)	BCRL (Affected)
1	128	47
2	96	23

**Table A3:** SIDE vs. BCRL contingency table.

SIDE	BCRL (Unaffected)	BCRL (Affected)
1	109	46
2	115	24

**Table A4:** TAXANE BASED CT vs. BCRL contingency table.

TAXANE BASED CT	BCRL (Unaffected)	BCRL (Affected)
0	121	22
1	103	48

**Table A5:** HT vs. BCRL contingency table.

HT	BCRL (Unaffected)	BCRL (Affected)
0	29	14
1	195	56

**Table A6:** LVI vs. BCRL contingency table.

LVI	BCRL (Unaffected)	BCRL (Affected)
0	153	36
1	71	34

**Table A7:** ECE vs. BCRL contingency table.

ECE	BCRL (Unaffected)	BCRL (Affected)
0	84	28
1	140	42

**Table A8:** ER vs. BCRL contingency table.

ER	BCRL (Unaffected)	BCRL (Affected)
0	26	11
1	198	59

**Table A9:** HER2 vs. BCRL contingency table.

HER2	BCRL (Unaffected)	BCRL (Affected)
0	206	56
1	18	14

**Table A10:** NCD vs. BCRL contingency table.

NCD	BCRL (Unaffected)	BCRL (Affected)
0	75	26
1	149	44

**Table A11:** PR vs. BCRL contingency table.

PR	BCRL (Unaffected)	BCRL (Affected)
0	40	21
1	184	49

**Table A12:** TTZ vs. BCRL contingency table.

TTZ	BCRL (Unaffected)	BCRL (Affected)
0	211	56
1	13	14

**Table A13:** Ki67 vs. BCRL contingency table.

Ki67	BCRL (Unaffected)	BCRL (Affected)
1	137	34
2	87	36

## Appendix C. Additional Tables for the Three Clusters Categorization

The following Table A14 summarizes the proportion of patients with or without BCRL inside each cluster, expecting a value of one in the case of equal groups.

**Table A14:** Ratio of BCRL patients vs. patients not affected by BCRL.

	Cluster A	Cluster C	Cluster B
BCRL	$\frac{4}{41}=0.097$	$\frac{16}{77}=0.207$	$\frac{50}{106}=0.471$

## Appendix D. Additional Tables for the Two Clusters Categorization

The association between the presence of BCRL and values of binary variables was studied using the Fisher exact test (in the case of frequency table entries of less than 5) or the $\chi^{2}$ test of independence on the 2×2 frequency table for all binary variables for each cluster. Only biomarkers that scored a p≤0.1 were included.

Only SIDE and TTZ binary variables were identified as not equally distributed (thus dependent) among patients of cluster B with the presence or not of BCRL (Table A15, significance level at p≤0.05). Variables LVI and HER2 were not far from significance, whereas all the other binary values can be considered independent of BCRL occurrence on both clusters.

**Table A15:** Association between binary variables and the presence of BCRL.

Variable	Cluster	Test Type	*p*
SIDE	B	$\chi^{2}$ test	0.0022
TTZ	B	$\chi^{2}$ test	0.0279
LVI	B	$\chi^{2}$ test	0.0628
HER2	B	$\chi^{2}$ test	0.0636

## Appendix E. Future Developments

Figure A1 outlined the entire analysis sequence for producing a predictive model using the labels generated by the UMAP clusters’ procedure demonstrated through the manuscript. The initial validation of the method was explained in Section 3.3, where the RF modeling of the dataset was nearly complete ensuring a solid foundation for future application of the machine learning algorithm to new data using three or two labels. In the future, the same classifier could be employed to score new patients and assign them a label associated with different BCRL risk profiles.
